# Diagnostic and therapeutic challenges of glioblastoma as an initial malignancy of constitutional mismatch repair deficiency (CMMRD): two case reports and a literature review

**DOI:** 10.1186/s12920-022-01403-9

**Published:** 2023-01-16

**Authors:** Shumpei Onishi, Fumiyuki Yamasaki, Kazuya Kuraoka, Akira Taguchi, Takeshi Takayasu, Kiwamu Akagi, Takao Hinoi

**Affiliations:** 1 Department of Neurosurgery, Graduate School of Biomedical and Health Sciences, Hiroshima University, Hiroshima, Japan; 2 Department of Neurosurgery, Kure Medical Center and Chugoku Cancer Center, National Hospital Organization, Hiroshima, Japan; 3 Department of Diagnostic Pathology, National Hospital Organization Kure Medical Center and Chugoku Cancer Center, Hiroshima, Japan; 4 Department of Molecular Diagnosis and Cancer Prevention, Saitama Cancer Center, Saitama, Japan; 5 Department of Clinical and Molecular Genetics, Hiroshima University Hospital, 1-2-3 Kasumi, Minami-Ku, Hiroshima, 734-8551 Japan

**Keywords:** Constitutional mismatch repair deficiency, CMMRD, Lynch syndrome, Glioblastoma, Colon cancer, Acute lymphocytic leukemia, PMS2, Genetic testing, Large deletion, Immune-checkpoint inhibitor

## Abstract

**Background:**

Constitutional mismatch repair deficiency (CMMRD) results from a biallelic germline pathogenic variant in a mismatch repair (MMR) gene. The most common CMMRD-associated malignancies are brain tumors; an accurate diagnosis is challenging when a malignant brain tumor is the only tumor at presentation. We describe two cases of glioblastoma as the initial CMMRD malignancy and discuss current diagnostic and therapeutic challenges.

**Case presentation:**

Two children with brain tumors without remarkable family history had biallelic pathogenic germline variants in *PMS2*. Patient 1: A 6-year-old girl presented biallelic *PMS2* germline pathogenic variants. Glioblastomas at the left frontal lobe and right temporal lobe were resistant to immune-checkpoint inhibitor, temozolomide, and bevacizumab. Patient 2: A 10-year-old boy presented biallelic *PMS2* germline variants. His glioblastoma with primitive neuroectodermal tumor-like features responded to chemoradiotherapy, but he developed advanced colon cancer and acute lymphocytic leukemia. In both patients, only a monoallelic *PMS2* germline variant was detected by conventional gene tests. PMS2 immunohistochemistry showed lack of staining at both the tumors and normal tissue as vascular endothelial cells. Further gene tests revealed large genomic deletion including the entire *PMS2* gene, confirming biallelic *PMS2* germline variants.

**Conclusion:**

Conventional multi-gene panel tests are insufficient for detecting large deletions of MMR genes, resulting in misdiagnoses of CMMRD as Lynch syndrome. *PMS2* variants have low cancer penetrance; family histories may thus be absent. Long-range gene analyses or immunohistochemical staining of MMR proteins in normal tissue should be considered for pediatric brain tumors with a single allele MMR variant when CMMRD is suspected.

**Supplementary Information:**

The online version contains supplementary material available at 10.1186/s12920-022-01403-9.

## Background

Constitutional mismatch repair deficiency (CMMRD) syndrome, a rare condition that greatly increases the risk of cancers among children, adolescents, and young adults [1], is caused by biallelic (homozygous or compound heterozygous) germline pathogenic variants in one of the mismatch repair (MMR) genes (*MLH1, MSH2, MSH6*, and *PMS2*). Monoallelic germline variants in MMR genes cause Lynch syndrome (LS) [2], which predisposes individuals mainly to colorectal cancer, endometrial cancer, and other LS-related malignancies. Individuals whose father and mother both have LS have a one-quarter risk of inheriting biallelic pathogenic variants of MMR genes that cause CMMRD.

Patients with CMMRD have presented malignancies including colorectal cancer, brain tumor, LS-related malignancy, or hematological malignancy [3]. Malignant brain tumors such as glioblastoma are the most common type of CMMRD-related malignancy. In the Care for CMMRD (C4CMMRD) database, 53.4% of the patients with CMMRD developed brain tumors, and the mean age at the brain tumor diagnosis is 9 years [3]. Glioblastomas that are associated with CMMRD have different oncogeneses, and they may represent a distinct entity of pediatric glioblastoma. Physicians thus need to discriminate CMMRD-associated glioblastomas from other pediatric high-grade gliomas (HGGs). In addition, a precise diagnosis of CMMRD has important implications for treatment and for the surveillance of the patients' families.

CMMRD can be challenging to diagnose in patients without a family history. Genetic screening for MMR gene variants is not routinely performed for brain tumors, and thus some CMMRD-associated malignant brain tumors may have been overlooked. Moreover, patients with CMMRD could be misdiagnosed as having Lynch syndrome, because the conventional multi-gene analyses are insufficient for detecting long-range deletions of MMR genes. In this report, we describe two cases of glioblastoma as the initial malignancy of CMMRD. In light of the results of conventional multi-gene analyses in both cases, there was a risk of potentially underestimating the gene variants as indicating Lynch syndrome.

## Case presentation

### Patient 1

**Fig. 1:**
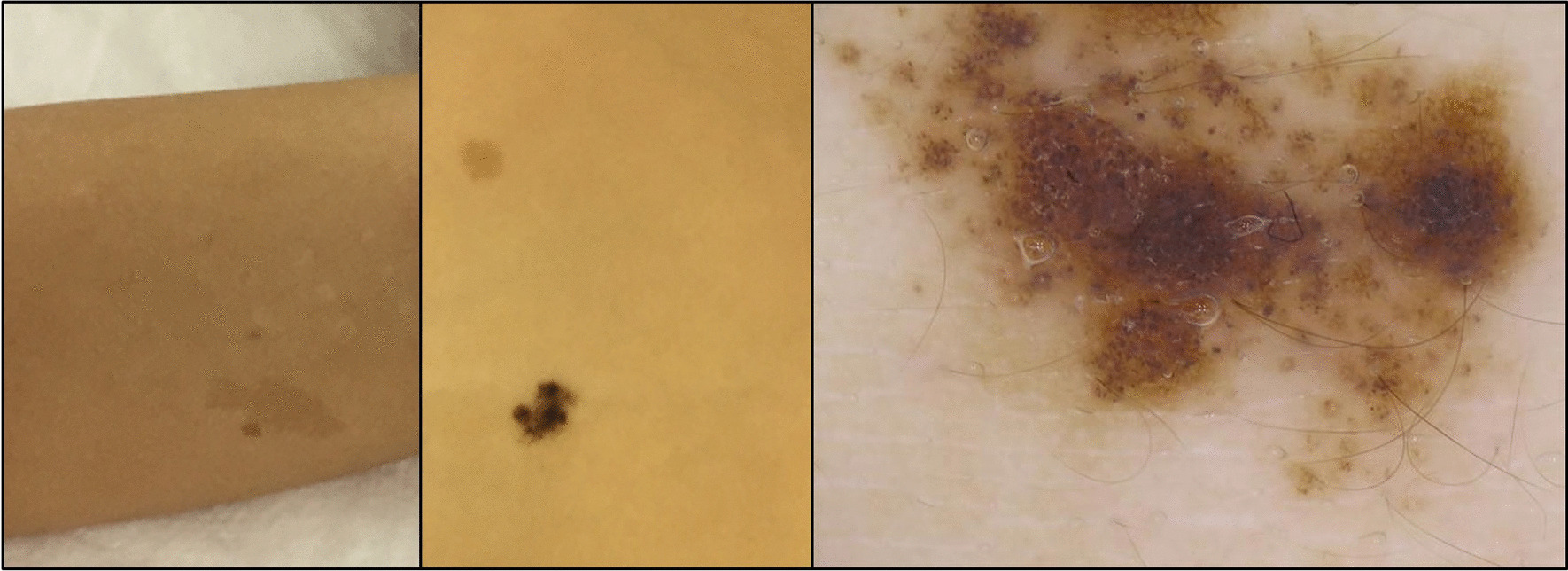
Multiple hyperpigmented skin alternations on the limbs and trunk of Patient 1, a 6-year-old girl

**Fig. 2:**
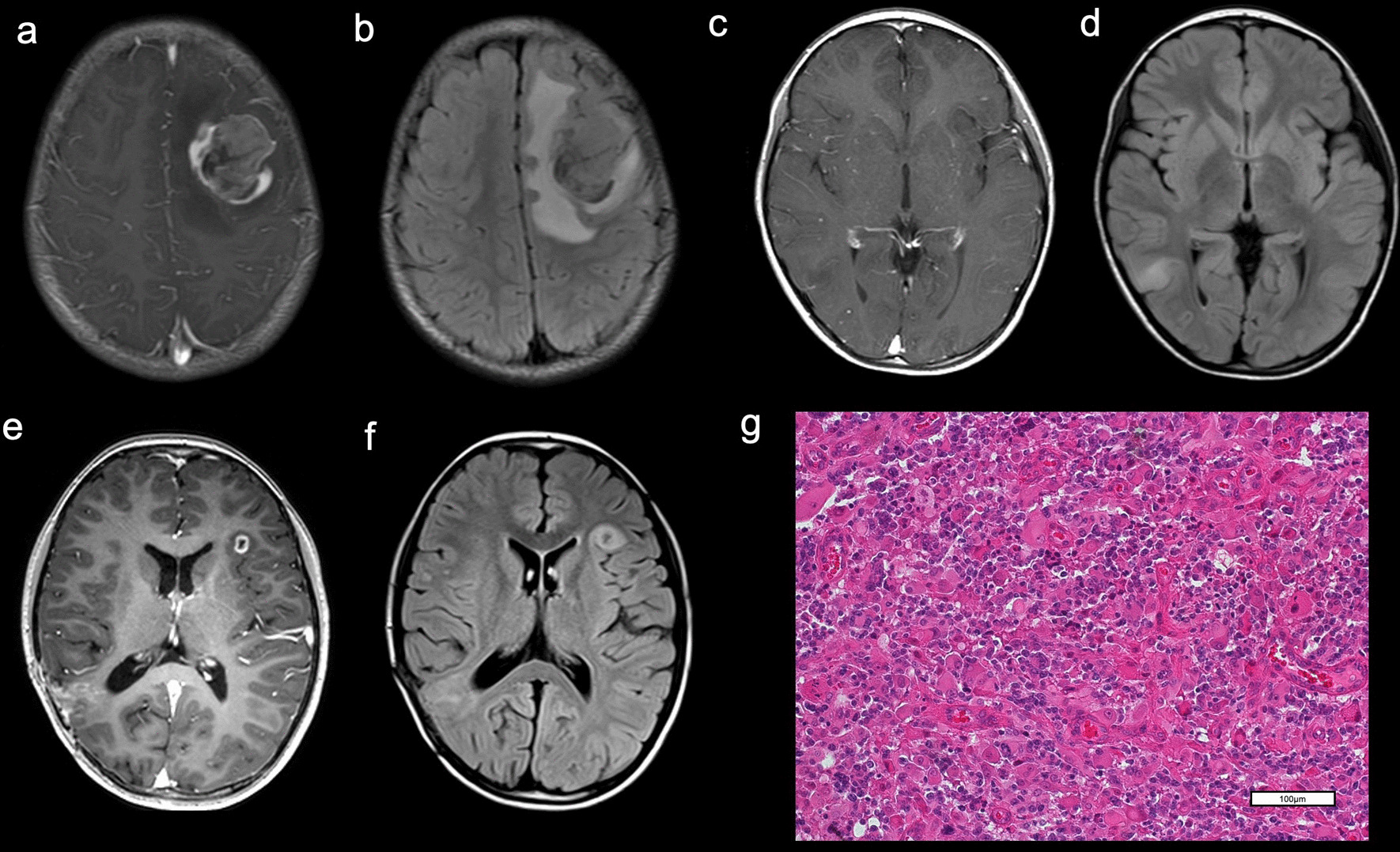
MRI of Patient 1 at onset showed a left frontal tumor. **a** The tumor was heterogeneously enhanced with gadolinium on T1WI. **b** FLAIR showed peritumoral edema. MRI **c, d** revealed that another non-enhancing tumor developed at the right temporal lobe. The tumor was hyper-intense on FLAIR **d** and was not enhancing with gadolinium on T1WI **c**. MRI **e, f** demonstrating a tumor at the left operculum. The tumor was enhanced with gadolinium on T1WI **e** and showed hyperintensity on FLAIR **f**. **g** Hematoxylin–eosin (HE) staining of a specimen from the 1st surgery for left frontal tumor showed a glioblastoma. (OLYMPUS BX43/ × 20 0.50FN 26.5, Nikon DIGITAL SIGHT DS-Fi2 Microscope C-mount Camera System, NIS ELEMENTS, resolution: 1280 × 960)

A six-year-old girl with no remarkable family history presented with headache and vomiting. She had hyperpigmented skin alternation, i.e., cafe au lait spots (Fig. 1). MRI showed a heterogeneous gadolinium-enhanced left frontal tumor (Fig. 2a, b). She underwent a left frontal craniotomy, and gross total removal of the tumor was achieved. Hematoxylin and eosin (HE) staining of the left frontal tumor showed glioblastoma with multinucleated giant cells (Fig. 2g). Molecular analyses by pyrosequencing showed no mutations in *IDH1/2*, *H3F3A, HIST1H3B*, or *BRAF*. The patient underwent standard local radiation therapy (RT): 60 Gy/30 fractions.

One year after the first surgery, another non-enhancing tumor developed at the right temporal lobe (Fig. 2c, d). This tumor was also completely resected. The pathological diagnosis was anaplastic astrocytoma, with the same molecular features as the initial glioblastoma. She underwent local RT: 50 Gy/25 fractions. The tumor later recurred at the right temporal tumor cavity, along with a new lesion at left operculum frontal lobe (Fig. 2e, f). These tumors were gross totally resected and carmustine wafers were implanted. The histological diagnosis of both tumors was glioblastoma. FoundationOne^®^ CDx (F1CDx) (Foundation Medicine, Cambridge, MA, USA) identified pathogenic variants in 30 genes (*POLE, ATM, HRAS, MET, NF1, PTEN, SMARCB1, STK11, ARID1A, RET, ATRX, CDKN1A, CIC, CSF1R, CTCF, DNMT3A, HSD3B1, KEL, KMT2A (MLL), MAP3K1, NOTCH1, NOTCH3, PBRM1, PMS2, PPP2R1A, RB1, RPTOR, STAG2, TP53, VHL*) and 92 variants of uncertain significance (Additional file 1: Table S1). The F1CDx test also revealed a high tumor mutation burden (TMB-H) of 192 mutations per megabase (muts/Mb), though designated the tumor as microsatellite stable (MSS). By Promega Microsatellite Instability (MSI) Analysis System (Madison, WI, USA), the tumor was designated microsatellite instability-high (MSI-H).

Immunohistochemical (IHC) staining of a specimen from the 1st surgery for the left frontal tumor showed a loss of PMS2 expression in tumor cells and normal tissue as vascular endothelial cells and preserved expression of MLH1, MSH2, and MSH6 in both tumor cells and normal tissue (Additional file 2: Figure S1a-d). Genetic testing revealed biallelic variants, i.e., *PMS2*:c.[241G > T];[2276-125_2445 + 1584del]. *PMS2*:c.241G > T was identified by the initial cancer genomic profiling test with F1CDx. However, another pathogenic variant, *PMS2*:c.2276-125_2445 + 1584del (which indicates the deletion of 1,879 base pairs [bps] including Exon 14 of *PMS2* gene, resulting in the stop codon in eight codons after codon 759 with frameshift mutation) was not detected by the F1CDx test, since the length of the deletion was too large to be detected by a next-generation sequencing (NGS) analysis in a cancer genomic profiling test. The large deletion was detected as described [4].

**Fig. 3:**
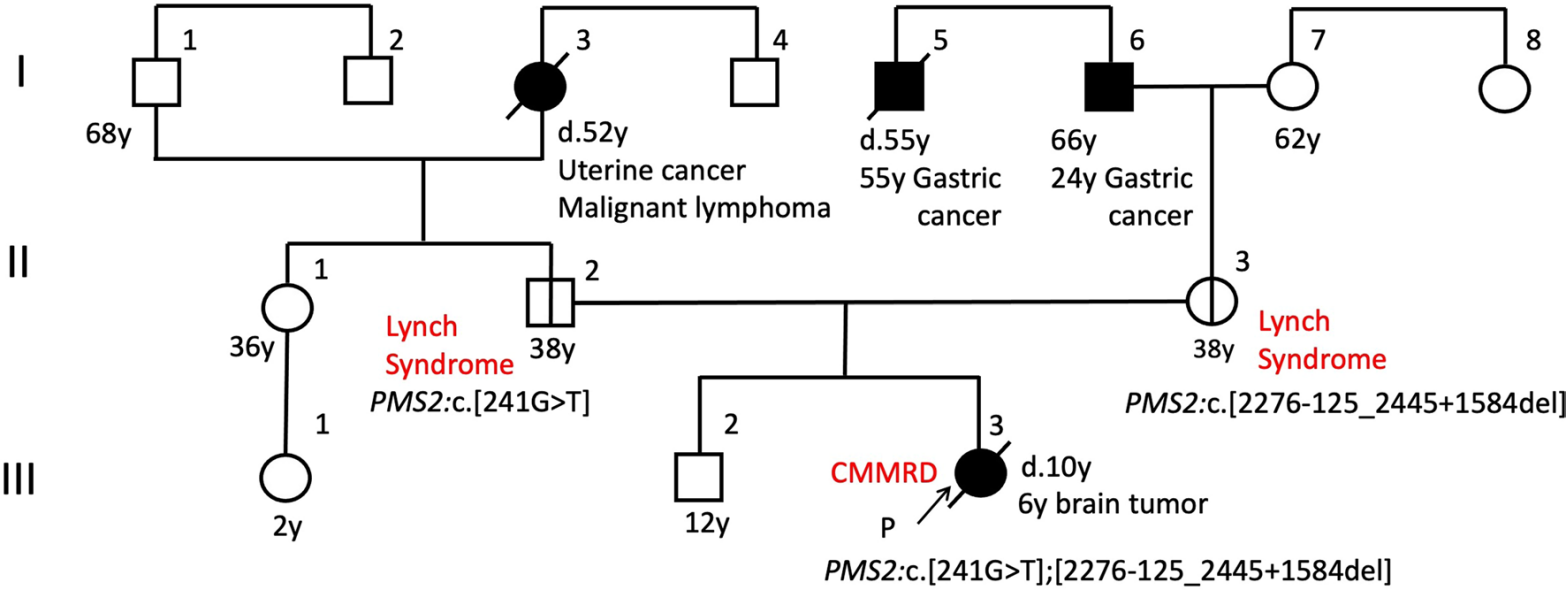
Pedigree showing affected and unaffected members of Patient 1's family

Parental genetic testing confirmed that the patient's father had one variant *PMS2*:c.241G > T, and the other variant, *PMS2*:c.2276-125_2445 + 1584del, was identified in the patient's mother. The interpretation of *PMS2*:c.241G > T in the ClinVar archive of the U.S. National Center for Biotechnology Information (ClinVar Variation ID: 439,243) is 'Pathogenic' and 'Likely pathogenic.' *PMS2*:c.2276-125_2445 + 1584del is registered as Class 5 (Pathogenic) in InSiGHT database of variants (https://insight-database.org/). A schematic image of the large deletion in *PMS2* gene is provided as Fig. 7. From this genetic information, Patient 1 was diagnosed with CMMRD, indicating compound heterozygous variants in *PMS2*, inherited from each of her parents (Fig. 3).

The tumors at the patient's right temporal lobe and left operculum frontal lobe recurred at tumor cavity walls. She was administered pembrolizumab every 3 weeks for three cycles, and this regimen was terminated after three cycles because of progressive disease with an increasing size of the tumor and the development of a new lesion at the left temporal lobe. She was subsequently administered temozolomide and bevacizumab; however, the tumor did not respond to these treatments, and the patient died 4 years after the initial surgery.

### Patient 2

**Fig. 4:**
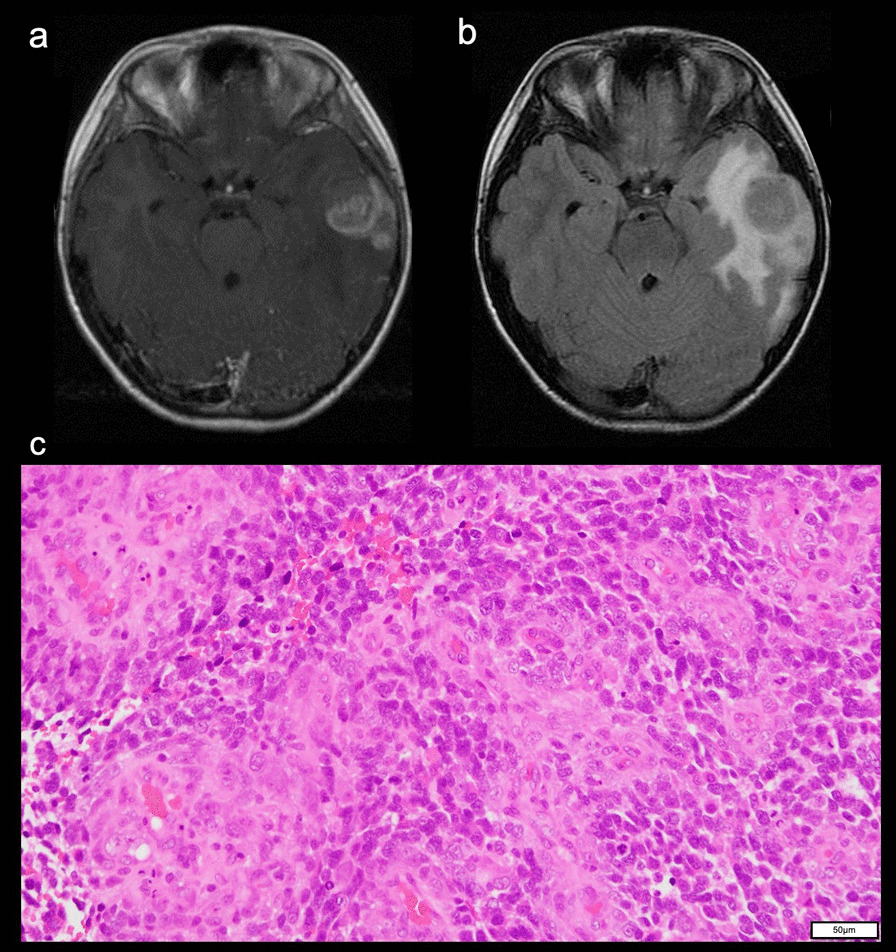
MRI of Patient 2, a 10-year-old boy at onset showing a left temporal tumor. **a** The tumor was heterogeneously enhanced with gadolinium on T1WI. **b** FLAIR showed peritumoral edema. **c**HE staining demonstrated a glioblastoma. (OLYMPUS BX53FZ/ × 20 0.50 FN 26.5, OLYMPUS DP 27, OLYMPUS Standard, resolution: 2448 × 1920)

A 10-year-old boy whose great-grandfather had colorectal cancer presented with a headache. His skin was not evaluated. MRI showed a gadolinium-enhanced left temporal tumor with peritumoral edema (Fig. 4a, b). The tumor was resected, and the histopathological diagnosis was glioblastoma with primitive neuroectodermal tumor-like features (Fig. 4c). The tumor was diagnosed with high-grade glioma, and the status of *IDH1/2, H3F3A, HIST1H3B* or *BRAF* was not analyzed.

Immunohistochemically, the tumor was positive for glial fibrillary acidic protein (GFAP). Immunohistochemical staining for MMR proteins showed a loss of PMS2 expression in tumor cells and vascular endothelial cells (Additional file 3: Figure S2a) and preserved expression of MLH1 (Additional file 3: Figure S2b), MSH2 (Additional file 3: Figure S2c) and MSH6 (Additional file 3: Figure S2d) in both tumor cells and vascular endothelial cells*.* The patient underwent craniospinal irradiation with local boost irradiation and eight cycles of platinum-based combination chemotherapy, and a complete response of the brain tumor was achieved.

**Fig. 5:**
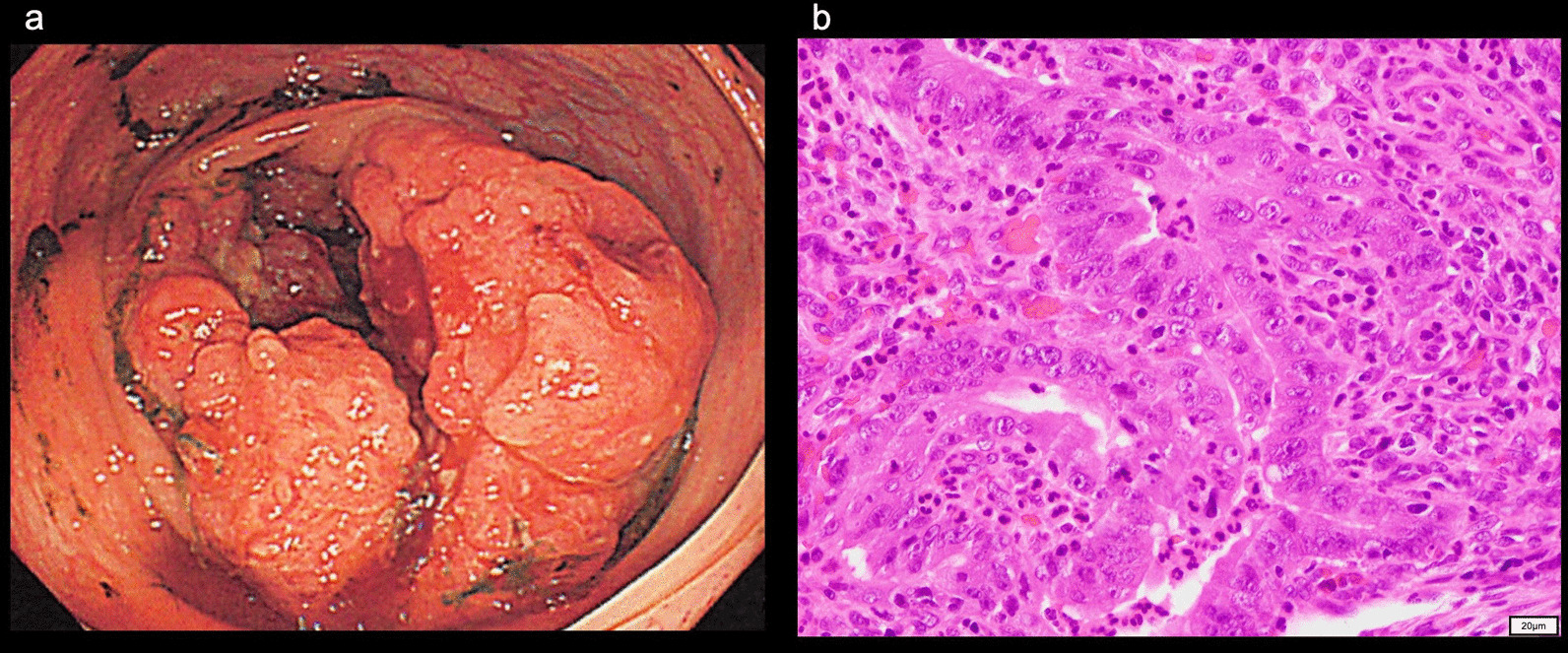
**a** Colonoscopy identified adenocarcinoma of the cecum. **b** HE staining showed a moderately differentiated tubular adenocarcinoma. (OLYMPUS BX53FZ / × 40 0.70FN 26.5, OLYMPUS DP 27, OLYMPUS Standard, resolution: 2448 × 1920)

Six years after the surgery, at the age of 16, the patient developed persistent abdominal pain. Colonoscopy revealed an adenocarcinoma of the cecum (Fig. 5a), and a right hemicolectomy with D3 lymph node dissection was performed. The diagnosis was moderately differentiated tubular adenocarcinoma (Fig. 5b), stage IIIb (pT3(A), ly3, v0, N2). After two cycles of chemotherapy with tegafur/uracil, the colon cancer recurred twice and the patient underwent local RT (60 Gy/20 fractions) each time to achieve the complete remission of the colon cancer.

Eight years after the brain tumor surgery, at 18 years old, the patient developed acute lymphocytic leukemia (ALL). He underwent chemotherapy based on the Japan Adult Leukemia Study Group (JALSG)-ALL202-U phase II multicenter study's schedule. Although a complete response of the ALL was obtained, colon cancer recurred, and mFOLFOX6 chemotherapy was administered.

IHC staining for MMR proteins of the patient's colon cancer revealed a loss of PMS2 in tumor cells and normal tissue as vascular endothelial cells and preserved expression of MLH1, MSH2, and MSH6 in both tumor cells and normal tissue (Additional file 4: Figure S3a-d). In addition, IHC staining of the initial brain tumor showed a loss of *PMS2* expression and preserved expression of MLH1, MSH2, and MSH6 (Additional file 3: Figure S2a-d).

**Fig. 6:**
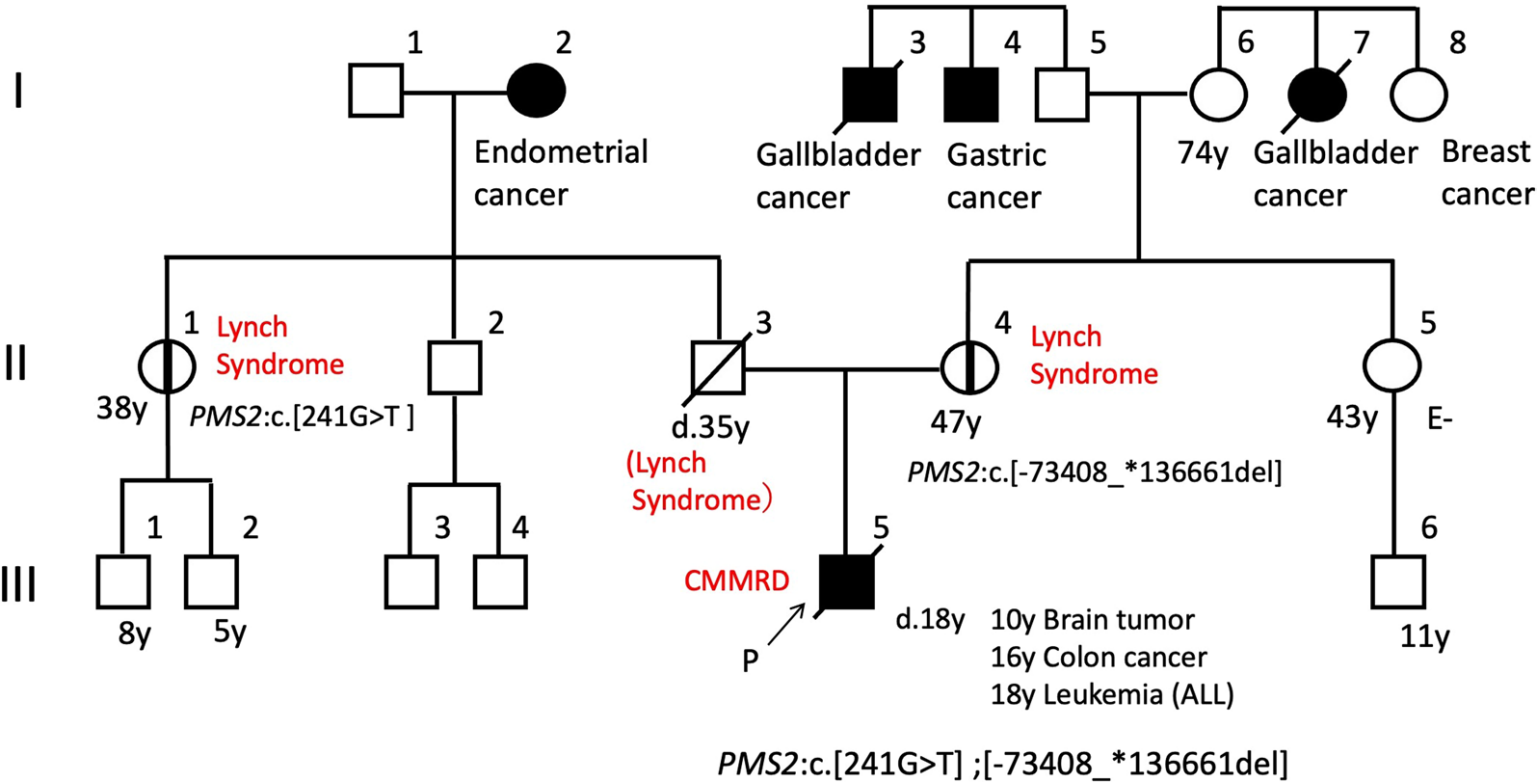
Pedigree showing affected and unaffected members of Patient 2's family

**Fig. 7:**
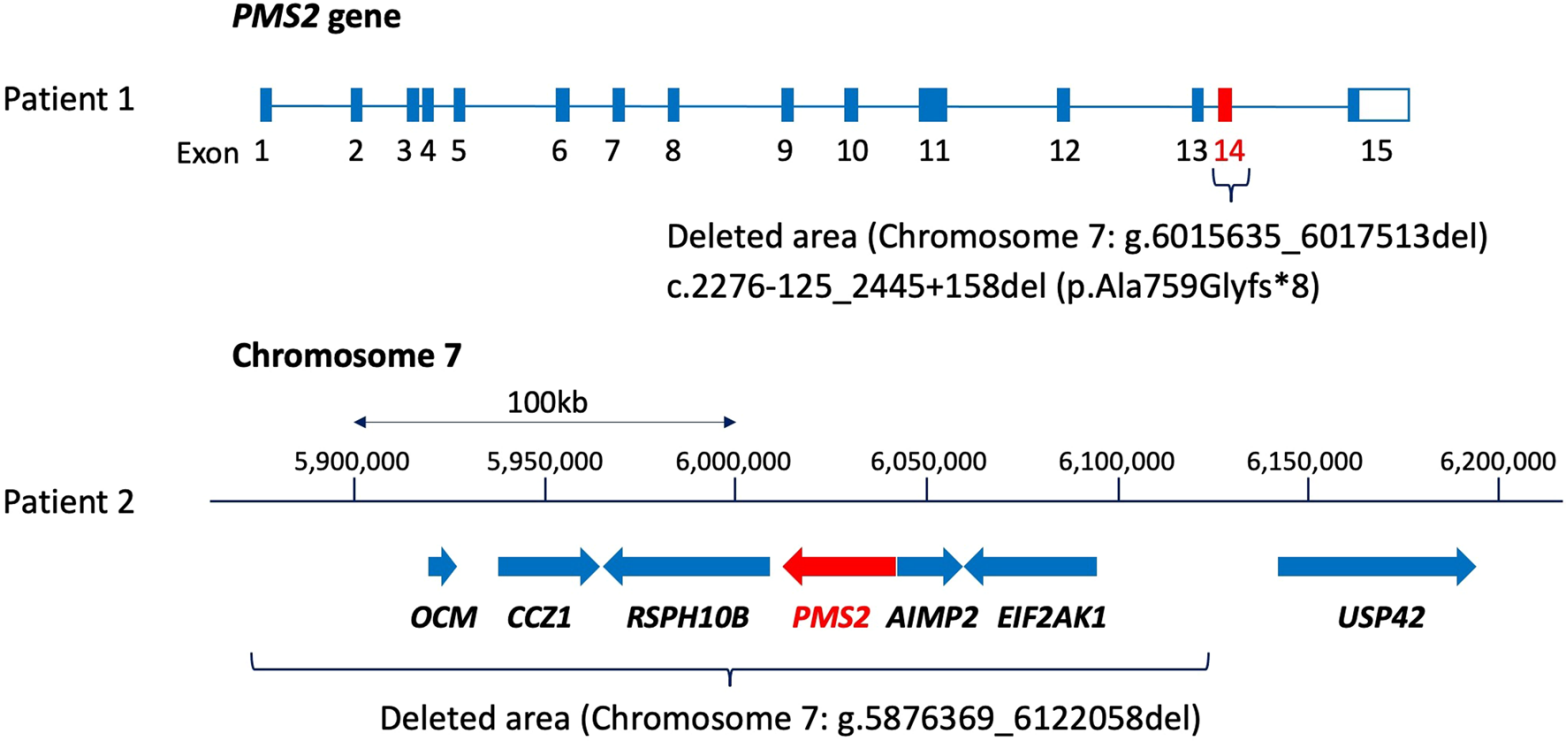
Schematic image of the large deletions in chromosome 7 and *PMS2* gene of Patients 1 and 2

A germline analysis using DNA and RNA extracted from the patient's peripheral blood as described [4] revealed biallelic *PMS2* variants, i.e., NC_000007.13 (chromosome 7): g.[5876369_612205del];[6043612C>A], which contains a large deletion including *PMS2*, and chromosome 7:g.5876369_612205del, which means PMS2:c.[−73408_*136661del], indicating the deletion of 245 kilo base pairs (kbps) including coding lesion of *PMS2*. Family genetic testing confirmed that the patient's paternal uncle had one variant, i.e., chromosome 7:g.6043612C>A, which means PMS2:c.241G>T, suggesting that the patient's father also carried the variant. The other variant, chromosome 7:g.5876369_6122058del, was identified in the patient's mother. The interpretation of *PMS2*:c.241G>T is 'Pathogenic' and 'Likely pathogenic' in ClinVar (ClinVar Variation ID: 439243). The other variant, chromosome 7: g.5876369_6122058del, caused the complete deletion of *PMS2* and five other genes, but the deletion was not registered in any database. Schematic images of large deletions in chromosome 7 are shown in Figure 7. From this genetic information, the diagnosis was CMMRD, indicating compound heterozygous variants in *PMS2*, inherited from each parent (Fig. 6).

Subsequently, the ALL recurred again without the recurrence of brain tumor or colon cancer, and the patient died 8 years after his initial brain tumor surgery.

## Discussion and conclusions

We have described the clinical pitfalls and diagnostic clues of CMMRD initially presenting as glioblastoma, and we presented two challenging cases of pediatric CMMRD-associated glioblastoma that were immune checkpoint inhibitor (ICI)-resistant, temozolomide-resistant, and bevacizumab-resistant. The precise diagnosis of CMMRD has important implications for the surveillance of patients' siblings and other family members. In Patient 1, metachronous HGG and cutaneous features were diagnostic clues. In a case series of CMMMRD-associated glioblastomas, 5 of 15 patients (33%) presented with metachronous brain lesions [5]. Skin alteration is also a characteristic of CMMRD. Most of the reported patients with CMMRD presented multiple café-au-lait maculae, which have a ragged edge and a slightly diffuse appearance [6]. The presence of HGG and multiple hyperpigmented skin alterations > 1 cm meet the indication criteria for CMMRD testing [3]. However, in the present Patient 2, CMMRD was diagnosed after he had developed three CMMRD-associated malignancies.

A comprehensive genomic profiling report of pediatric HGG revealed that 6% (9/157) of pediatric HGGs were hypermutated, and most of them harbored pathogenic variants in one of the MMR genes [7]. Among sporadic pediatric HGG patients evaluated in Jordan, MMRD was observed in 39% (17/44), and 82% (14/17) of these were biallelic MMRD [8]. These results implied that CMMRD may be overlooked in current clinical practice for pediatric HGG.

In the CMMRD database, *PMS2* gene is also the most common causative gene of CMMRD-associated brain tumors, accounting for 60% of CMMRD patients [3]. However, *PMS2* variants have low penetrance [9], and they may lack a family history of Lynch syndrome-spectrum malignancies. Therefore, the Amsterdam I/II and revised Bethesda guidelines could be insufficient for screening for Lynch syndrome [10], especially for the *PMS2* variant. The estimated prevalence of Lynch syndrome in general populations is approx. one in 370 [11], and CMMRD could be underdiagnosed. Clinicians should consider the possibility of CMMRD when a brain tumor is detected, because a malignant brain tumor could develop as an initial presentation of CMMRD syndrome.

Biallelic MMR deficiency would be detected in a CMMRD-associated tumor; however, conventional multi-gene panel tests are not able to detect a large deletion of MMR genes. In Patient 1, the initial result of a multi-gene panel test (the F1CDx test) was a single monoallelic *PMS2* variation, c.241G > T (p.Glu81*). The analysis pipeline of the F1CDx test can detect the short abnormal structure of the targeted gene, but it cannot detect a large deletion of the targeted gene. Another *PMS2* variant, the deletion of 1,879 bps including Exon 14, was too large to be detected in conventional multi-gene panel tests. In Patient 2, chromosome 7:g.5876369_612205del indicates the deletion of 245 kbps caused all coding region of *PMS2* with five other exons. The extremely large deletion including the entire *PMS2* gene was undetectable in conventional multi-gene panel tests. Physicians may therefore misdiagnose CMMRD patients as having Lynch syndrome when only conventional multi-gene panel tests are used. Even if a pathogenic variant in one of the MMR genes is detected by a conventional analysis, the potential existence of another MMR pathogenic variant should be carefully examined by other methods such as a long-range analysis around MMR genes, as described [4].

Immunohistochemistry staining of MMR proteins in normal tissue could also be valuable for further investigations of MMR genes in clinical practice. IHC staining is a cost-effective strategy for screening MMR deficiency. MMR proteins are normally present in human cells. In LS patients, the expression of certain MMR proteins is lost in tumor cells and retained in normal cells such as endothelial cells. In CMMRD patients, the expression is lost in tumor and normal cells. The loss of one or more MMR proteins by IHC staining in 'normal tissues' thus suggests biallelic germline pathogenic variants of MMR gene [12]. The normal brain tissue surrounding a brain tumor should thus be carefully observed.

TMB-H and neoantigen loads have been suggested to be associated with the efficacy of ICIs [13], and based on these characteristics, ICIs are considered an effective therapy for CMMRD-associated glioblastoma. Several CMMRD-associated glioblastomas reportedly showed a significant response to ICI treatment [14, 15]. However, the experience of Patient 1 did not support the efficacy of ICI treatment for recurrent CMMRD-associated glioblastomas with TMB-H. There have been no clinical trials showing the benefit of an ICI in adjuvant therapy for glioblastomas [16], but the addition of an ICI as neoadjuvant treatment indicated more consistent immune activation and efficacy for recurrent glioblastoma [17]. To improve the treatment of CMMRD-associated glioblastoma, further evidence is necessary to establish predictive markers for the efficacy of ICIs and to identify the best treatment schedule including neoadjuvant settings.

The ICI therapies for MSI-H malignancies have shown promising results in some cancers. MSI testing with the conventional multi-gene panel tests (e.g., F1CDx) may lead to discrepancies in companion diagnostics using Bethesda and Promega panels. The advantage of a comprehensive genomic profiling (CGP) test is the simultaneous analysis of the MSI status, genomic aberrations, and the TMB in solid tumors. A cohort study that used the F1CDx test for diffuse glioma reported that most of the glioblastoma indicated MSS despite a high TMB score [18]. However, another study demonstrated that the accuracy of an NGS-based MSI analysis is not perfect [19]. The Promega MSI Analysis System is considered the gold-standard panel for MSI detection in cancers because of its higher sensitivity and specificity according to the revised Bethesda guidelines for colorectal cancers [20, 21]. In the present two patients, the results of the F1CDx microsatellite test were MSS, whereas the Promega MSI Analysis System revealed MSI-H in both patients. MSI testing should be performed by multiple methods if MSI-H is suspected based on other findings.

In conclusion, the accurate and early diagnosis of CMMRD has important implications for the management of the patients and their families. Our report highlights the shortcomings of conventional multi-gene panel tests for diagnosing CMMRD and microsatellite instability, which might have resulted in an underestimation of the incidence of CMMRD among pediatric patients with malignant brain tumors. Clinicians should consider conducting a long-range gene analysis and/or IHC staining of MMR proteins in normal tissue for pediatric patients with brain tumors and a single allele MMR variant for the diagnosis of CMMMRD.

## Supplementary Information

**Additional file 1: **The list of pathogenic variants and variants of uncertain significance (VUS) detected in glioblastoma of patient 1 by FoundationOne® CDx test.

**Additional file 2: Fig. S1**. Immunohistochemical staining for MMR proteins showed a loss of PMS2 expression in tumor cells and normal tissue as vascular endothelial cells (a) and preserved expressions of MLH1 (b), MSH2 (c) and MSH6 (d) in both tumor cells and vascular endothelial cells. White arrows indicate the nucleus of the vascular endothelial cells (a-d). (OLYMPUS BX43/×40 0.70FN 26.5, Nikon DIGITAL SIGHT DS-Fi2 Microscope C-mount Camera System, NIS ELEMENTS, resolution: 1280 × 960)

**Additional file 3: Fig. S2** IHC staining for MMR proteins showed a loss of PMS2 expression in tumor cells and normal tissue as vascular endothelial cells (a) and preserved expressions of MLH1 (b), MSH2 (c) and MSH6 (d) in both tumor cells and vascular endothelial cells. (OLYMPUS BX53FZ/×20 0.50 FN 26.5, OLYMPUS DP 27, OLYMPUS Standard, resolution: 1224 x 960)

**Additional file 4: Fig. S3** IHC staining for MMR proteins showed a loss of PMS2 in tumor cells and normal tissue (c) and preserved expressions of MLH1 (d), MSH2 (e) and MSH6 (f) in both tumor cells and normal tissue. (OLYMPUS BX53FZ /×40 0.70FN 26.5, OLYMPUS DP 27, OLYMPUS Standard, resolution: 1224 x 960)

## Data Availability

The details of the variant analyzed in this study have been deposited into the ClinVar database (https://www.ncbi.nlm.nih.gov/clinvar/), under the accession number SCV002600096, SCV002600097 and SCV002600098.

## Abbreviations

bps: Base pairs
CGP: Comprehensive genomic profiling
CMMRD: Constitutional mismatch repair deficiency
F1CDx: FoundationOne^®^ CDx
GFAP: Glial fibrillary acidic protein
HGG: High-grade glioma
ICI: Immune checkpoint inhibitor
IHC: Immunohistochemical
LS: Lynch syndrome
MMR: Mismatch repair
MSI: Microsatellite instability
MSS: Microsatellite stable
NGS: Next-generation sequencing
TMB-H: High tumor mutation burden
