# Supplementary figures and images for: Diagnostic and therapeutic challenges of glioblastoma as an initial malignancy of constitutional mismatch repair deficiency (CMMRD): two case reports and a literature review
Source: BMC Med Genomics. 2023 Jan 16;16:6. doi: 10.1186/s12920-022-01403-9 (PMC9843912; doi:10.1186/s12920-022-01403-9)

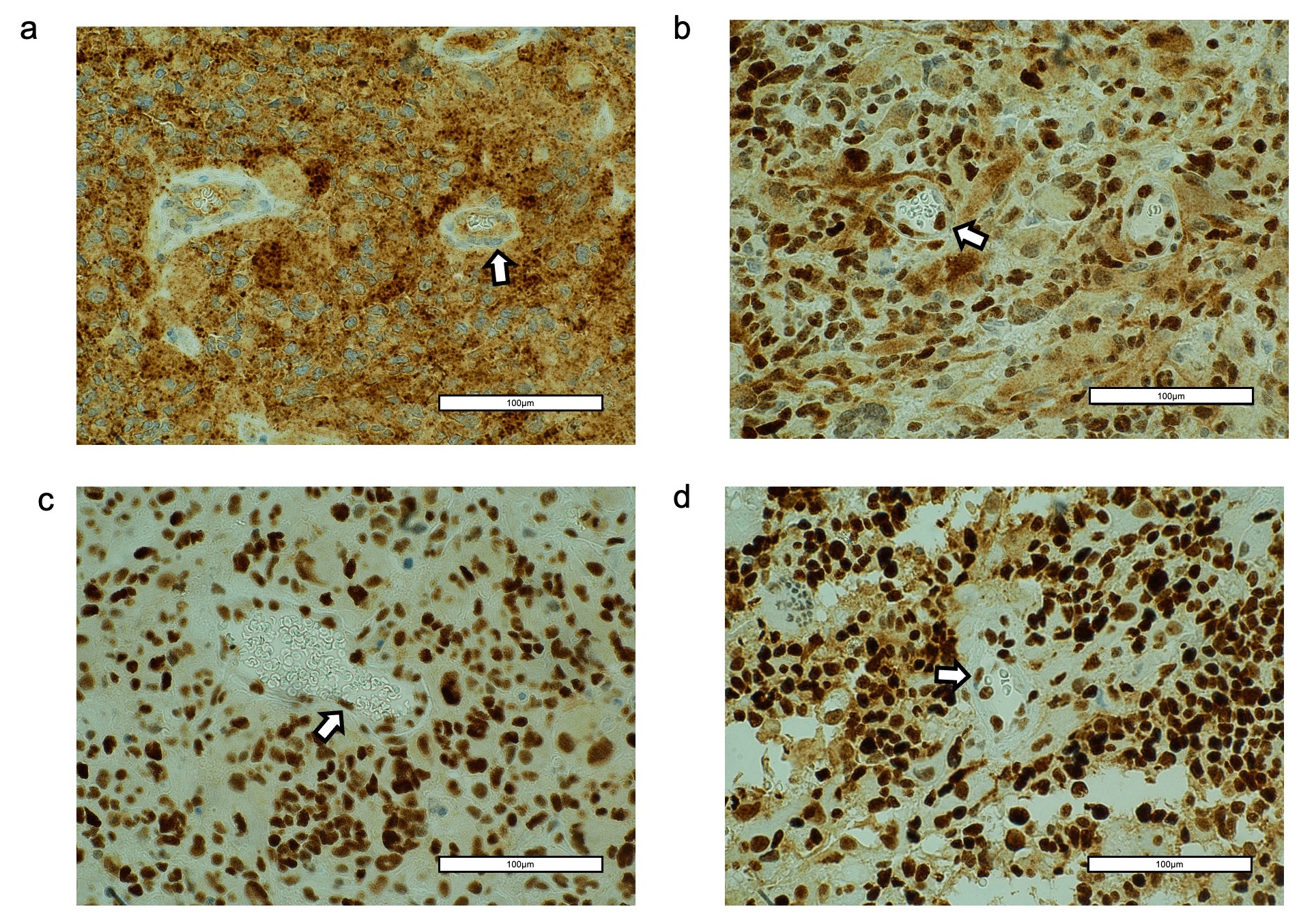

Supplement: Supplementary file 2 — Additional file 2: Fig. S1. Immunohistochemical staining for MMR proteins showed a loss of PMS2 expression in tumor cells and normal tissue as vascular endothelial cells (a) and preserved expressions of MLH1 (b), MSH2 (c) and MSH6 (d) in both tumor cells and vascular endothelial cells. White arrows indicate the nucleus of the vascular endothelial cells (a-d). (OLYMPUS BX43/×40 0.70FN 26.5, Nikon DIGITAL SIGHT DS-Fi2 Microscope C-mount Camera System, NIS ELEMENTS, resolution: 1280 × 960) [file 12920_2022_1403_MOESM2_ESM.jpeg]

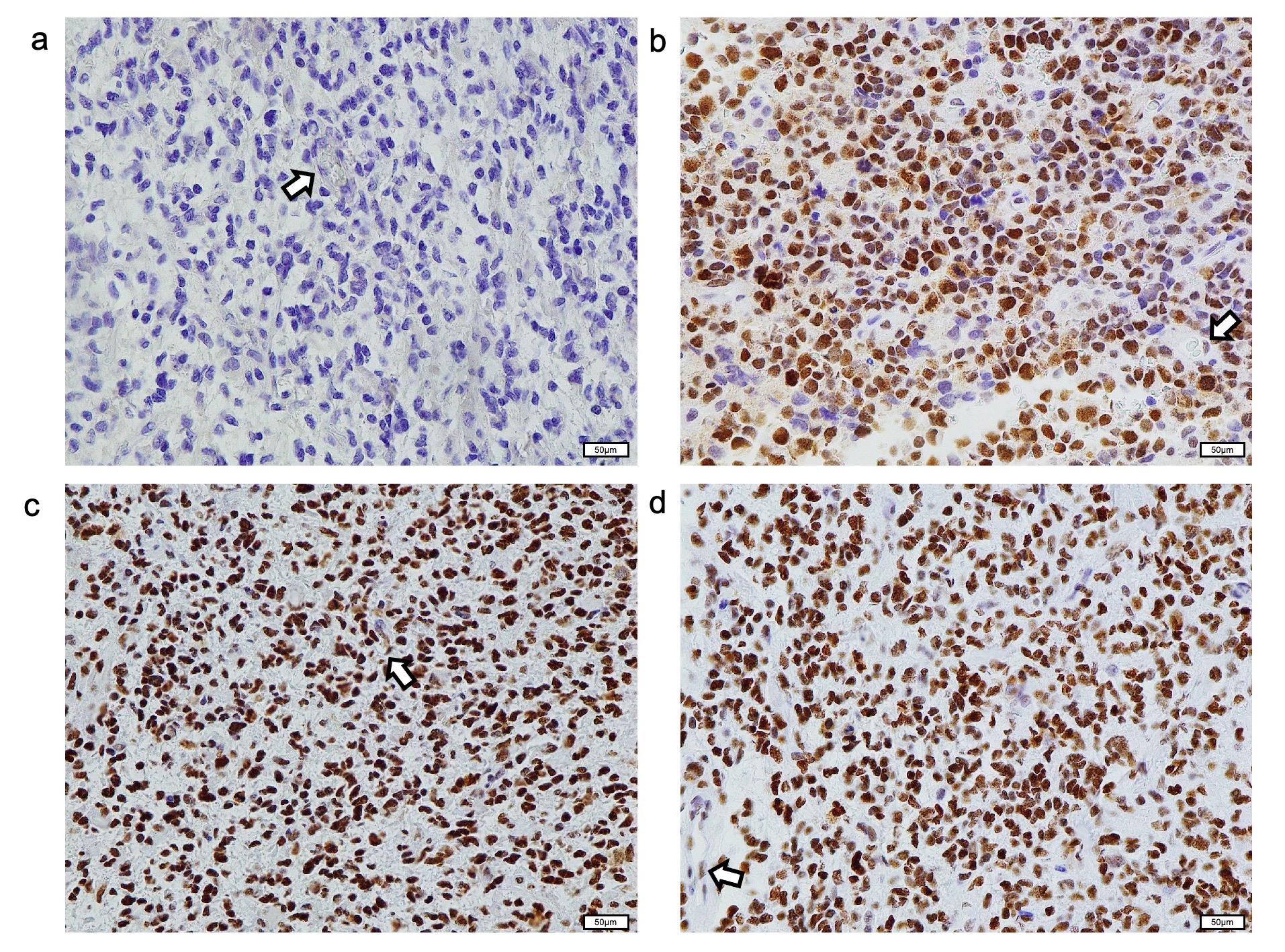

Supplement: Supplementary file 3 — Additional file 3: Fig. S2 IHC staining for MMR proteins showed a loss of PMS2 expression in tumor cells and normal tissue as vascular endothelial cells (a) and preserved expressions of MLH1 (b), MSH2 (c) and MSH6 (d) in both tumor cells and vascular endothelial cells. (OLYMPUS BX53FZ/×20 0.50 FN 26.5, OLYMPUS DP 27, OLYMPUS Standard, resolution: 1224 x 960) [file 12920_2022_1403_MOESM3_ESM.jpg]

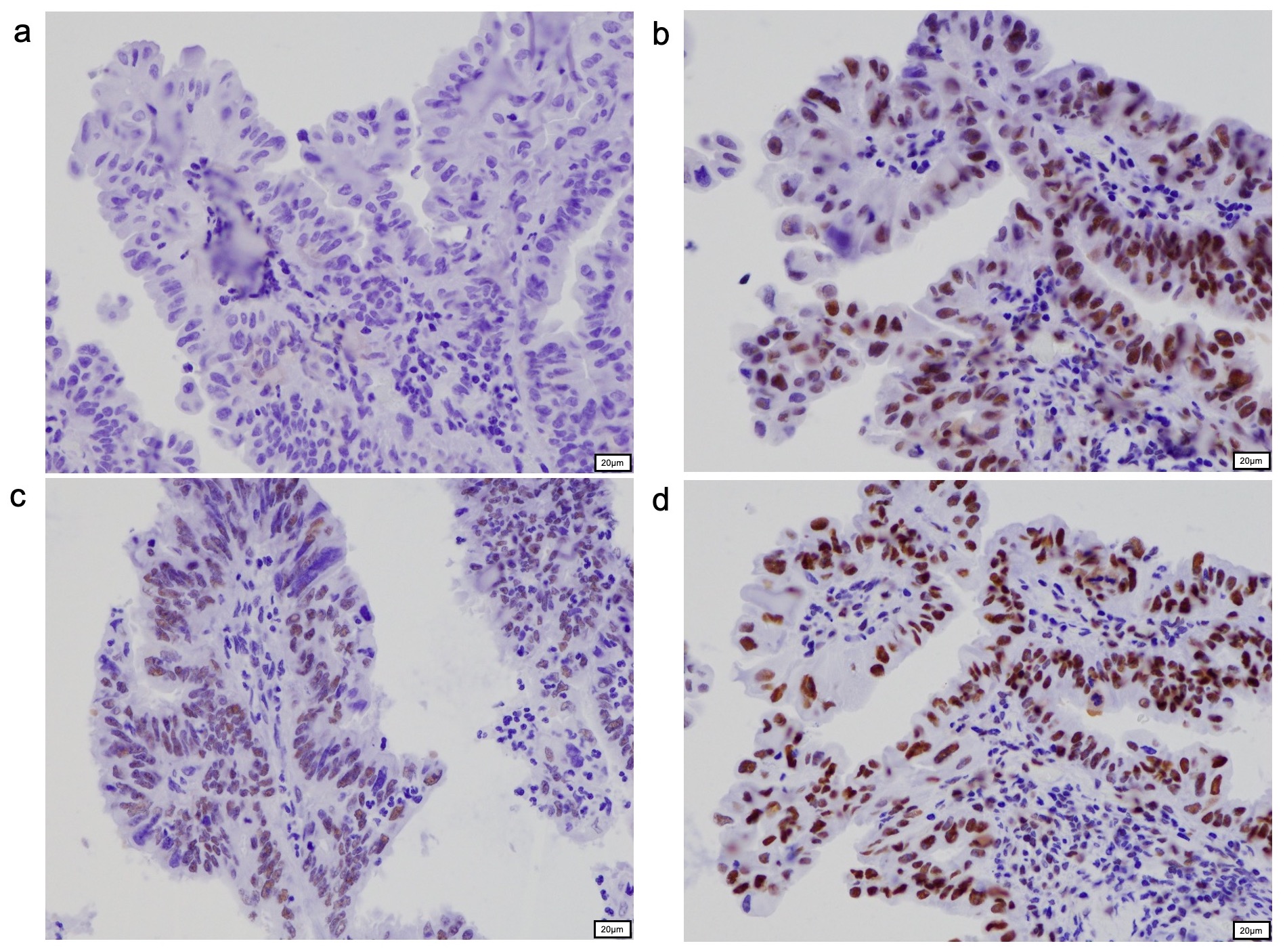

Supplement: Supplementary file 4 — Additional file 4: Fig. S3 IHC staining for MMR proteins showed a loss of PMS2 in tumor cells and normal tissue (c) and preserved expressions of MLH1 (d), MSH2 (e) and MSH6 (f) in both tumor cells and normal tissue. (OLYMPUS BX53FZ /×40 0.70FN 26.5, OLYMPUS DP 27, OLYMPUS Standard, resolution: 1224 x 960) [file 12920_2022_1403_MOESM4_ESM.jpeg]
